# Negative Energy Balance Does Not Alter Fat-Free Mass During the Yukon Arctic Ultra—The Longest and the Coldest Ultramarathon

**DOI:** 10.3389/fphys.2018.01761

**Published:** 2018-12-20

**Authors:** Adriane Schalt, Michelle M. Johannsen, Jimin Kim, Richard Chen, Carl J. Murphy, Melynda S. Coker, Hanns-Christian Gunga, Robert H. Coker, Mathias Steinach

**Affiliations:** ^1^Charité - Universitätsmedizin Berlin, Institute of Physiology, Center for Space Medicine and Extreme Environments Berlin, Berlin, Germany; ^2^Institute of Arctic Biology, University of Alaska Fairbanks, Fairbanks, AK, United States

**Keywords:** body composition, cold exposure, cytokines, extreme environment, ultramarathon

## Abstract

**Purpose:** The objective of this study was to determine alterations in caloric balance, body composition, metabolites, and cytokines in athletes participating in the Yukon Arctic Ultra.

**Methods:** Ten participants traveling on foot in the 2017 692-km event were recruited for the study. Measurements and samples were obtained at pre-event, 278 km (C1), 384 km (C2), and post-event. Body composition measurements were obtained using bioelectrical impedance analysis. Accelerometer devices were utilized to provide an estimation of caloric expenditure and dietary recalls provided assessments of caloric intake. Blood serum samples were collected, processed, and analyzed using enzyme-linked immunosorbent assays or nuclear magnetic resonance. Results were analyzed using linear mixed model, presented as means ± SD, and considered significant at *p* < 0.05.

**Results:** Participants (8 males, 2 females; age: 37 ± 10 years; body mass index: 24.4 ± 2.5 kg/m^2^) were recruited. Four males and one female completed the entire event in 260 ± 19 h. Caloric intake/expenditure was 4,126 ± 1,115 kcal/day and 6,387 ± 781 kcal/day, respectively, indicating a caloric deficit of 2,261 ± 1,543 kcal/day. Total mass, body mass index, and fat mass were reduced at each time point of the event. Fat-free mass (FFM) was unchanged throughout the event. Follistatin was increased at C1 (1,715 ± 876 pg/ml) in comparison to baseline. Acetoacetate increased significantly at post-event (6.1 ± 1.5 mg/ml).

**Conclusions:** Despite a pronounced caloric deficit and sustained activity under extreme cold conditions, FFM was preserved with an increase in serum follistatin and acetoacetate. Future studies should be directed at the role of nutrient strategies and/or training methods on the retention of FFM under these conditions.

## Introduction

The Yukon Arctic Ultra (YAU) has been advertised as the “World’s Toughest and Coldest Ultra” (Montane, 2017). Participants must travel 692 kilometers (430 miles) from Whitehorse to Dawson City, Yukon Territory. The event takes place in the winter months when temperatures can easily reach as low as −48°C. Historical data show individuals average a speed of 3–5 km/h, sleep only 2–6 h per night, and it typically takes participants 7–12 days to complete the event. Due to the strenuous nature of the YAU, it is not uncommon for 50% or more of the individuals to drop out of the event. Rigorous safety standards may also disqualify individuals from continuing if officials deem that it is unsafe (e.g., signs of frostbite).

Over 20 years ago, the physiological resilience of two men who walked 2,300 km across Antarctica completely unsupported was described (Stroud et al., 1996). Utilizing a high-fat diet, these individuals adequate to elevated levels of protein synthesis that fostered the preservation of lean tissue (Stroud et al., 1996). In previous studies with the athletes participating in the YAU, we evaluated changes in body composition, serum cytokines, and metabolites (Coker et al., 2017). Remarkably, fat mass (FM) was reduced but fat-free mass (FFM) was preserved in a very limited number of participants with only one athlete completing the entire event (Coker et al., 2017). Additional studies performed with athletes in the Alaska Mountain Wilderness Ski Classic under similar Arctic winter conditions also demonstrated the preservation of lean tissue mass despite high levels of energy expenditure, as measured by dual-energy X-ray absorptiometry (Johannsen et al., 2018). While all of these data are interesting (Stroud et al., 1996; Coker et al., 2017; Johannsen et al., 2018), alterations in FM relative to FFM in conjunction with the estimation of caloric balance during exercise under chronic, extreme cold conditions have not been thoroughly investigated.

Myostatin and follistatin have been posited to exert a direct role in skeletal muscle growth and proliferation (Lee et al., 2010; Bragg et al., 2014). Studies have described the short-term influence of modest exercise and/or limited cold exposure on these glycoproteins (Wolfe et al., 1984; Haman, 2006). For example, myostatin mRNA is suppressed by cold exposure (Ijiri et al., 2009) and exercise (Zak et al., 2018). Given that myostatin plays an inhibitory role in muscle growth (Bragg et al., 2014) and follistatin may enhance muscle growth (Lee et al., 2010), it is important to evaluate whether they may be altered during the YAU or similar events/scenarios.

The objective of the present study was to determine the impact of the participation in the YAU on: (a) caloric balance and metabolites, (b) body composition, and (c) follistatin and myostatin that have not been comprehensively evaluated under these conditions. We hypothesized that participants would sustain a chronic net negative caloric balance, develop a metabolite profile indicative of ketogenesis, and maintain FFM. We further hypothesized that myostatin would decrease, and follistatin would increase in conjunction with the retention of FFM. The mechanisms responsible for survival in the cold environment are heavily dependent upon the preservation of lean tissue mass (Haman 2006; Haman and Blondin, 2017). Therefore, it is important to completely understand these mechanisms that might be applied to circumstances involving military or space personnel, bush pilots, and other minimally supported individuals who may face similar occupational conditions. The loss of FFM is also important in space exploration as the dietary intake of astronauts falls below positive caloric balance and further increases the loss of FFM, and may compromise the overall mission (Stein et al., 1999).

## Materials and Methods

### General Information

Participants of the 2017 YAU 692-km (430-mile) event were recruited for this study (n = 10, 8 males, 2 females; 37 ± 10 years of age). Nine of the participants were of Caucasian descent, one of Asian descent. Five were able to finish the complete distance (one female, four males). All subjects gave their informed written consent to partake in the study. The study was approved by the Charité Ethics Board (review number EA4/109/12), and all procedures complied with the Declaration of Helsinki (54th Revision 2008, Korea) regarding the treatment of human subjects.

These individuals were at chronic risk for the development of frostbite and/or hypothermia. First-time athletes were required to complete a training course that detailed first aid, shelter, equipment, nutrition, trails, and other necessary information necessary to sufficiently prepare the individuals for the arduous conditions. All competitors were required to pull their own sleds to carry equipment. Vital equipment included food, water, clothing, cooking utensils, sleeping preparations, and emergency preparedness items. In spite of these precautions, 50% of the study participants in 2017 were unable to complete the race for a variety of reasons ranging from severe muscle cramps to frostbite. We added an additional checkpoint in comparison to our work on the 2015 event so that data and samples were collected at pre-event (Whitehorse), checkpoint 1 (C1) at 278 km (Carmacks), checkpoint 2 (C2) at 384 km (Pelly Crossing), and post-event (Dawson City).

### Body Composition

Body composition was measured using bioelectrical impedance analysis (BIA) *via* the tetrapolar electrode method (Sun et al., 2003), which is an established method to determine body composition (Gudivaka et al., 1999). These measurements were taken with an Akern BIA 101 (Florence, Italy) at the four checkpoints of the event: pre-event, C1, C2, and post-event. The BIA measurements provided values for FFM, and total body water (TBW) using equations appropriate for this cohort:

FFM Males (kg) = 9.33285 + 0.0006636 Height (cm)^2^ − 0.02117 Resistance (Ω) + 0.62854 Weight (kg) − 0.1238 Age (years);

FFM Females (kg) = 10.43485 + 0.00064602 Height (cm)^2^ − 0.01397 Resistance (Ω) + 0.42087 Weight (kg) (Segal et al., 1988).

TBW Males (l) = 1.203 + 0.499 Height (cm)^2^/Resistance (Ω) + 0.176 Weight (kg);

TBW Females (l) = 3.747 + 0.45 Height (cm)^2^/Resistance (Ω) + 0.113 Weight (kg) (Sun et al., 2003).

FM was calculated by substracting FFM from total mass (TM). Fat-free mass index (FFMI) was calculated as (FFM/height^2^), as was fat mass index (FMI), respectively (FM/height^2^). At each of the checkpoints, measurements were conducted indoors under similar room temperature conditions, just after awakening from an overnight rest, with a voided bladder, prior to breakfast, and dressed with light underwear.

### Dietary Intake

Dietary recall information was obtained from the participants *via* a “Food & Energy Intake Form” and confirmed by a nutritional specialist. Participants were required to fill out the form in advance, naming the food items and including information such as kcals, weight, quantity packed, and quantity consumed. The caloric content of meals prepared by race organizers at the checkpoints was estimated using the type of dish on the menu as reference. Estimation of caloric content from the completed forms was based on information provided in “Food and Nutrition Tables” (Souci et al., 2016). All values were then verified by a professional dietitian from the Charité Health Academy.

### Energy Balance

Energy expenditure was estimated using the SenseWear Pro3 Armband (Bodymedia, Pittsburgh, PA). The SenseWear Professional software was used to analyze and intepret the raw data. (King et al., 2004; Welk et al., 2007; Almeida et al., 2011). This particular device has been demonstrated to provide a reliable and accurate method for the measurement of physical activity and the assessment of energy expenditure (King et al., 2004; Welk et al., 2007; Koehler and Drenowatz, 2017). Data for dietary recall and energy expenditure were used to calculate net caloric balance for the participants.

### Blood Parameters

Blood serum samples were collected by a physician under consistent room temperature conditions at each checkpoint and centrifuged to separate whole blood from serum. Serum samples were then pipetted into cryovials and stored in a liquid nitrogen dewar for transport at −80°C and later analysis.

Myostatin and follistatin were measured by enzyme-linked immunosorbent assay according to manufacturers’ instructions (R & D systems, Minneapolis, MN). All samples were analyzed in triplicate. Serum samples for NMR-derived metabolite analysis were thawed and vortexed to ensure homogeneity and transferred into 5-mm NMR tubes (Wilmad Lab Glass, Buena, NJ). ^1^H–NMR spectra were acquired at 17°C (based on methanol calibration) with a 600-MHz Bruker Avance-III system running TopSpin 3.2 software (Bruker Biospin, Fremont, CA), and using a dual-resonance high-resolution SmartProbe with single axis Z-gradient (Nicholson et al., 1995). The water signal was suppressed using NOESY presaturation followed by CPMG relaxation method, editing for suppression of macromolecules (“PROF_CPMG” parameter set in TopSpin 3.2). A standard, trimethylsilyl propionic-2,2,3,3-tetradeuteropropionic acid (TMSP, 3.87 mM in D_2_O) contained in a sealed insert and placed in the NMR tube was used for metabolite quantification of fully relaxed ^1^H–NMR spectra and as a ^1^H chemical shift reference (0.0 ppm). After Fourier transformation, phasing, and baseline correction in TopSpin, each ^1^H peak was integrated (Bogren et al., 2014). The ^1^H-NMR peaks for single metabolites were identified and referred to published chemical shift or a metabolite chemical shift library. The absolute concentration of each metabolite was then referred to the TMSP integral and calculated according to the equation: *C_x_*  =  (*I_x_*/*N_x _*·*C*)/*I/*9, where *Cx* is the metabolite concentration (μmol/ml), *Ix* is the integral of metabolite ^1^H peak, *Nx* is the number of protons in metabolite ^1^H peak, *C* is the TMSP concentration, and *I* is the integral of TMSP ^1^H peak at 0 ppm (this is nine as TMSP contains nine protons) (Serkova et al., 2005). An additional correction factor of 11.304 was applied to adjust for the differences in diameters between the NMR tube and the insert (determined using reference samples). The coefficient of variation for alanine, fatty acids, lactate, acetoacetate, ß-glucose, histidine, and formate was 0.2, 0.3, 0.4, 0.5, 0.4, 0.1, and 0.7, respectively. The final metabolite concentrations were expressed as mg/ml.

Statistical analysis was performed using R Studio version 1.1.422 software. Since 50% of the participants were unable to complete the event, missing data points existed and data were analyzed using a linear mixed model approach. The mixed-effect approach works in essence the same as a traditional ANOVA, but accounts for missing data as well as the repeated measures study design when determining if differences exist between the four time points of the event (Gelman and Hill, 2007; Mallinckrod et al., 2008). This allows for the use of our entire sample of participants, and avoids arbitrary selection and the potential for the biased interpretation of our data. A *post hoc* analysis using the Tukey’s honest significant difference test was then used to determine if differences were statistically significant (Gelman and Hill, 2007; Mallinckrod et al., 2008). Values are reported as means ± SD and were considered significant with a *p* < 0.05.

## Results

### Characteristics

Eight males and two females were initially recruited for the study (*n* = 10; age = 37 ± 10 years). Due to the harsh environmental conditions, only 5 of the 10 participants were able to complete the 2017 event. One male and one female dropped out before C1. By C2, two males had dropped out. Between C2 and the finish, another male dropped out of the event. Of the five participants that were able to complete the event, the average completion time was 260 ± 19 h. Therefore, four males and one female completed the entire event.

### Body Composition

Total body mass (TM) and FM were reduced at each time point (Table 1). There were no significant changes in body mass index (BMI), FFM, or FFMI (Table 1).

**Table 1:** Body composition.

	Pre-event (*n* = 8)	Checkpoint 1 (*n* = 8)	Checkpoint 2 (*n* = 6)	Post-event (*n* = 5)
BMI (kg/m^2^)	24.3 ± 2.9	23.7 ± 2.4	23.8 ± 3.0	22.8 ± 2.7
TM (kg)	75.9 ± 12.1	74.0 ± 10.5*	75.7 ± 10.9	70.7 ± 9.3*
FFM (kg)	62.6 ± 9.8	62.0 ± 9.2	62.8 ± 9.4	59.3 ± 7.4
FM (kg)	13.3 ± 3.2	12.0 ± 2.6*	12.9 ± 2.6	11.4 ± 2.9*
FM (%)	17.5 ± 2.6	16.2 ± 2.8*	17.1 ± 2.6	16.1 ± 2.8*
FFMI (kg/m^2^)	20.1 ± 2.3	19.9 ± 2.0	19.7 ± 2.4	19.7 ± 2.2
FMI (kg/m^2^)	4.3 ± 0.9	3.9 ± 0.8*	4.1 ± 0.9	3.7 ± 1.0*
TBW (liters)	44.6 ± 5.2	46.5 ± 5.4	47.2 ± 4.5	46.9 ± 2.6

Data are presented as mean ± SD.

* Different from previous time point. p < 0.05.

### Hydration Status

Total body water (TBW) remained stable at each time point (Table 1).

### Caloric Balance

Complete dietary recall data were provided by all five participants who finished the event. The average caloric intake for these participants was 4,126 ± 1,115 kcals/day. Their corresponding average energy expenditure was 6,387 ± 781 kcals/day, indicating a negative caloric balance of 2,261 ± 1,543 kcals/day.

### Metabolites

Fatty acids and derivatives and alanine and ß-glucose were decreased initially at C1 and remained reduced throughout the event (Table 2). There was a transient decrease in serum lactate at C1 and C2 and a reduction in serum histidine at (C2) (Table 2). Serum acetoacetate was increased substantially at post-event (Table 2).

**Table 2:** Alterations in serum metabolites.

Metabolite	Pre-event (*n* = 8)	Checkpoint 1 (*n* = 8)	Checkpoint 2 (*n* = 6)	Post-event (*n* = 5)
Alanine (mg/mL)	5.21 ± 0.34	3.63 ± 0.29*	3.48 ± 0.15*	3.28 ± 0.29*
Fatty Acids (mg/mL)	8.24 ± 1.62	3.81 ± 0.41*	4.45 ± 0.64	4.09 ± 0.69*
Lactate (mg/mL)	37.33 ± 5.38	20.76 ± 1.92*	23.43 ± 2.03*	26.39 ± 3.60
Acetoacetate (mg/mL)	0.82 ± 0.13	3.08 ± 1.08	2.97 ± 0.67	6.09 ± 1.56*
ß-glucose (mg/mL)	13.47 ± 1.52	20.35 ± 1.82*	18.14 ± 1.12*	19.03 ± 2.27*
Histidine (mg/mL)	1.00 ± 0.05	0.80 ± 0.046	0.71 ± 0.06*	0.80 ± 0.12
Formate (mg/mL)	0.07 ± 0.02	0.13 ± 0.01*	0.13 ± 0.02*	0.12 ± 0.03

Data are presented as means ± SD.

* Different from pre-event value. *p* < 0.05.

### Cytokines

**Figure 1:**
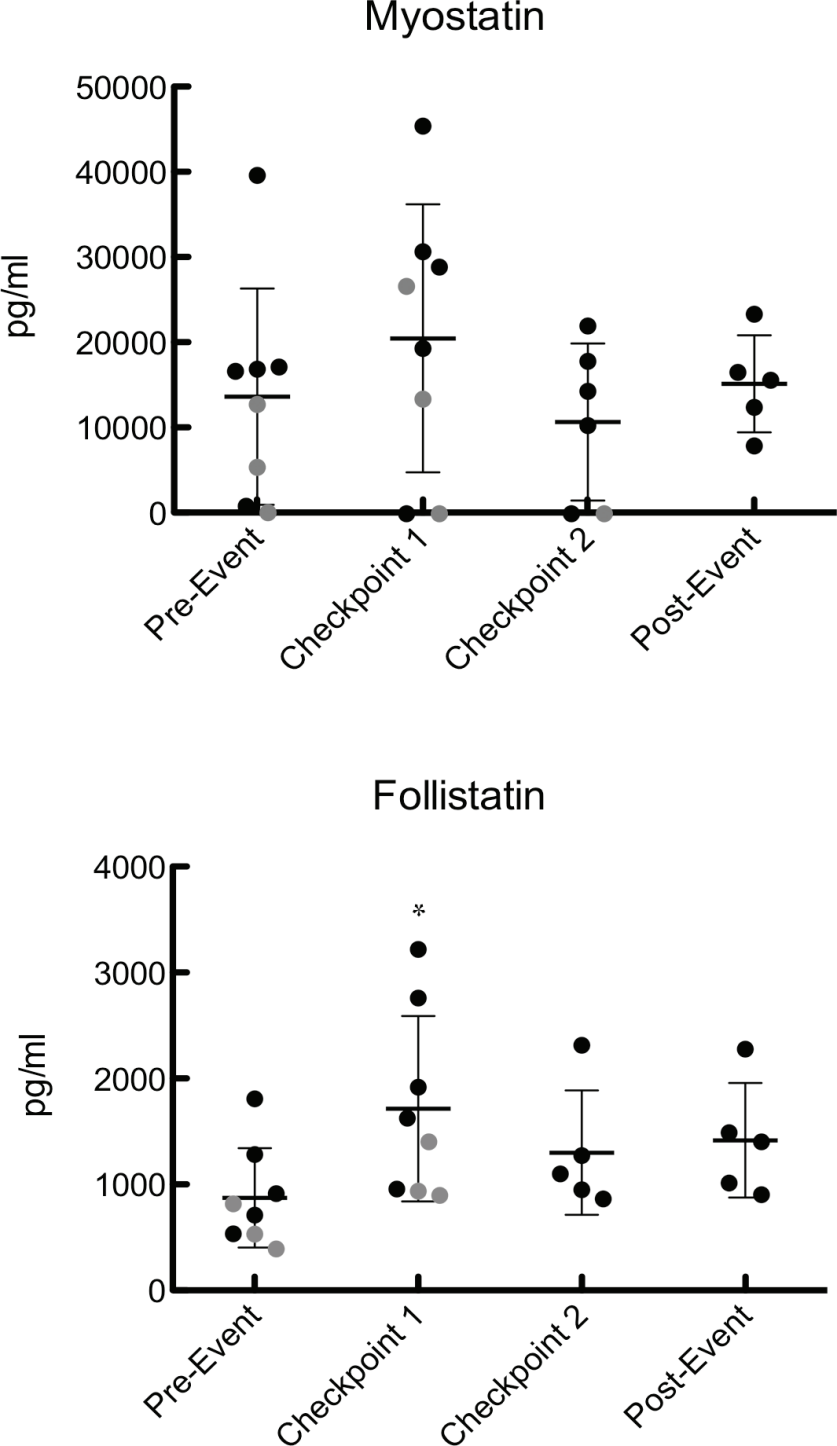
Serum follistatin and myostatin during pre-event, C1, C2, and post-event. Data points colored in black represent finishers and data points represented in gray represent dropouts. For samples below the limit of detection of the assay (270 pg/ml for myostatin), the samples were assigned a value of half the LOD to avoid biasing the results. Values are expressed as mean ± SD. *Represents significant difference from baseline.

Blood serum was analyzed for myostatin and follistatin concentrations at each of the aforementioned time points (Figure 1). There were no significant changes in myostatin (pre-event: 13,676 ± 12,617 pg/ml, C1: 20,528 ± 15,595 pg/ml, C2: 10,656 ± 8,915 pg/ml, post-event: 15,172 ± 5,669 pg/ml), but the values at C1 and C2 were below detectable limits for one participant (*p* = 0.35) (Figure 1). Follistatin was significantly higher at C1 than any other time point of the event (pre-event: 871 ± 470 pg/ml, C1: 1,715 ± 876 pg/ml, C2: 1,355 ± 544 pg/ml, post-event: 1,417 ± 542 pg/ml) (Figure 1).

## Discussion

The primary objective of this study was directed at the assessment of caloric intake and caloric expenditure, body composition, and alterations in serum metabolites in athletes traversing the entire 692-km distance of the YAU. We were also interested in potential alterations in myostatin and follistain in conjunction with this event. Similar to our previous investigations in athletes exercising in the extreme cold for extended periods (Coker et al., 2017; Johannsen et al., 2018), there was a sustained amount of negative energy balance, significant reductions in FM but no change in FFM. Exceptionally challenging cold conditions in the 2017 YAU were noted, considerably worse during the first half of the event, and this made food preparation extremely difficult. In spite of this situation, energy intake was quite high at over 4,000 kcal/day. These findings seem to suggest that successful athletes may utilize a combination of training, nutrient, and pacing strategies that enable adaptive responses to the physical, psychological, and environmental stresses of the event.

Energy balance plays a significant role in the modulation of protein turnover and maintenance of FFM (Pasiakos et al., 2010), and the severity of negative energy balance results in detrimental reductions in physical performance (Murphy et al., 2018). In an example of sustained negative energy balance from physical exertion and dietary restriction over an extended period (−1,000 kcal/day over an 8-week period), the investigators reported simultaneous muscle atrophy and decreased physical function (Nindl et al., 2007). This work highlights one of the more dramatic scenarios describing negative caloric balance over a significant period of time. Moreover, the interactive influence of dietary restriction and physical exertion across the duration of this type of physiological stress plays a major role in the risk of muscle atrophy (Tassone and Baker, 2017). It is unclear whether the duration of these physiological challenges emerged as a primary factor in the deterioration of physical function.

Recent research has shown that participants of ultraendurance events tend to maintain FFM even with the added stress of cold exposure (Stroud et al., 1996; Saugy et al., 2013; Coker et al., 2017; Johannsen et al., 2018). Caloric expenditure and intake were not examined in conjunction with body composition analysis in our previous work with this particular cohort (Coker et al., 2017). We have now demonstrated that athletes were able to maintain FFM even with an estimated caloric deficit of approximately 2,200 kcals/day. While this may seem quite remarkable, pre-clinical studies have highlighted the interorgan transfer of amino acids that may contribute to the conservation of muscle and other tissues, even during short-term exercise (Williams et al., 1996). This assertion has been further supported by the results of clinical studies that utilized stable isotope methodology to demonstrate that protein degradation was not increased by acute exercise and the diversion of amino acids toward the exercised muscle (Devlin et al., 1990).

In addition to the influence of exercise on the sequestration of amino acids by the working muscle (Devlin et al., 1990), nutrient intake and/or progressive, exertion-linked changes in fuel selection may play a significant role in the sparing of amino acids that facilitate the maintenance of FFM (Burke, 2015). Macronutrient intake data were not available in this particular study, but similar expeditions have reported variable dietary intake of approximately ~35–66% carbohydrate, 29–55% fat, and 17–20% protein in ultra-athletes (Wolfe et al., 1984; Stroud et al., 1993, 1996; Gannon et al., 2001; Praz et al., 2015). We know from our own anecdotal observations during the 2015 and 2017 YAU that many of these athletes may consume more fat than described in earlier studies. This seems plausible, as these particular athletes were required to carry the majority of their dietary provisions in their pulks during the event and dietary fat represents a more efficient method of caloric delivery relative to the amount of weight pulled. In our study, plasma acetoacetate increased progressively, eightfold relative to baseline by the end of the event. This indicates an increasing reliance on ketones as a fuel during prolonged exercise (Galvin et al., 1968) and that these YAU participants may modify their use of fat as fuel (Pitsiladis et al., 1999) through potentially beneficial responses in PGC1α mRNA (Slivka et al., 2012). Reductions in lactate and alanine suggest a gluconeogenic shift in the provision of circulating glucose, but they are not conclusive due to the inability to assess glucose kinetics in this environmental setting (Coker and Kjœr, 2005). Future studies in this cohort will focus on whether athletes “train” their ability to convert to ketosis and/or the molecular adaptations that play a role in their resilience.

We measured myostatin and follistatin because of their widely known roles in regulating muscle growth and metabolism (Lee et al., 2010; Bragg et al., 2014; Druet et al., 2014), and yet limited information with regard to their adaptive responses to sustained exertion and cold exposure in a “real-world” field setting. It has been demonstrated that these cytokines are influenced by factors other than exercise including cold exposure, diet, and sleep deprivation (Coker and Kjœr, 2005; Allen et al., 2011; Hansen et al., 2011; Vamvini et al., 2011). Previous work by Zak et al. (2018) also demonstrated rapid alterations in myostatin mRNA in response to exercise performed even under moderately cold conditions. It is highly likely that participants in this event experienced all of these physiological stressors and to a greater absolute and relative extent than comparisons made to well-controlled laboratory conditions. While the primary mediators responsible for alterations in these cytokines remain unclear, numerous factors play a role in protein metabolism under conditions of physiological stress. For example, interleukin-6 has been also demonstrated to initially increase during strenuous exercise, promote alterations in substrate utilization that could be beneficial to skeletal muscle (Pedersen et al., 2001), and then decline in a fashion similar to the alterations in follistatin.

One of the limitations of our study involves the use of the SenseWear device to estimate energy expenditure. The device may underestimate energy expenditure during exercise performed at a high intensity (i.e., >10 METs) (Drenowatz and Eisen, 2011). However, due to the nature of the event lasting over 10 days, it is quite likely that the predominance of physical exertion ranged only from low to moderate exercise intensity. Therefore, our results are very likely consistent with other studies that have demonstrated the SenseWear device as a reliable method for the estimation of energy expenditure in a variety of settings (King et al., 2004; Welk et al., 2007; Koehler and Drenowatz, 2017). We also recognize that the presentation of dietary intake data that only includes an estimation of energy intake without information about macronutrient intake may be somewhat limiting. Many ultramarathon athletes may manipulate macronutrient intake such that the fat intake increases in proportion to carbohydrate and protein (Peters, 2003). This may have a beneficial influence on fat oxidation (Leckey, 2018), but the lack of macronutrient data in this study limits our ability to provide inferences in this regard. Furthermore, we acknowledge the limitations of the single-frequency bioimpedance analysis used in this investigation to estimate FM, FFM, and TBW. BIA represents an indirect method to estimate body composition that is not only dependent on the appropriate regression formula but also on procedural consistency of its application. In addition, alterations among various cohorts may also distort the results, for example, kidney diseases, cardiovascular conditions, etc., as well as ethnic group affiliation (Kyle et al., 2004). However, we have employed formulas appropriate for this cohort of healthy trained adults to estimate FM and FFM (Segal et al., 1988) and TBW (Sun et al., 2003) that have been shown to perform better than other formulas with smaller biases and better agreement (Seoane et al., 2015). In addition, great care was taken to perform the measurements under the same conditions by the same operator.

In this study, we were able to extend our previous findings suggesting the preservation of FFM during the Yukon Arctic Ultra. These findings are limited by the use of methodologies such as the estimation of caloric balance and body composition by indirect assessment. Serum concentrations of metabolites and progressively increasing levels of acetoacetate posit alterations in metabolic regulation of a potential shift toward and increasing reliance on ketones as fuel. We have also demonstrated that follistatin increases initially and then returns to baseline. Future studies should be directed at the assessment of macronutrient intake and additional cytokines that may be involved in the maintenance of physiological resilience throughout the event.

## Author Contributions

MJ assisted in sample transport, conducted analysis on cytokines and metabolites, and contributed to manuscript; AS conducted research, recruited participants, collected all samples, calculated energy expenditure and energy intake, and contributed to the manuscript; JK and RC conducted analysis on cytokines and contributed to manuscript; CM conducted analysis on metabolites and was responsible for the oversight of molecular analysis; MC assisted in sample transport and contributed to the manuscript; H-CG contributed to research plan and manuscript; RC contributed to research plan and cytokine analysis, was responsible for sample transport, and the final draft of the manuscript; MS contributed to research plan, recruited participants, collected all samples, served as study physician, calculated energy expenditure and energy intake, and contributed to the manuscript.

### Conflict of Interest Statement

Dr. Coker is a managing partner and co-owner of Essential Blends, LLC that has received funding from the National Institutes of Health to develop clinical nutrition products. The data presented in this manuscript are unrelated. We declare that the results of the study are presented clearly, honestly, and without fabrication, falsification, or inappropriate data manipulation.
